# Expression of Concern: Protection of LPS-induced intestinal injury in goslings by polysaccharide of *Atractylodes macrocephala* Koidz based on 16S rRNA and metabolomics analysis

**DOI:** 10.1371/journal.pone.0355346

**Published:** 2026-08-05

**Authors:** The PLOS One Editors

After this article [1] was published, the following concerns were noted:

- The articles cited as References 23 and 44 were retracted after [1] was published.

In addition, the *PLOS One* Editors have concerns about the article’s compliance with the PLOS Authorship policy.

In light of these issues, the *PLOS One* Editors issue this Expression of Concern. Readers are advised to interpret the article [1] with caution.
