# Supplementary material for: Protection of LPS-induced intestinal injury in goslings by polysaccharide of Atractylodes macrocephala Koidz based on 16S rRNA and metabolomics analysis
Source: PLoS One. 2025 Jun 18;20(6):e0326164. doi: 10.1371/journal.pone.0326164 (PMC12176298; doi:10.1371/journal.pone.0326164)
Supplement: Attachment — Submitted filename: renamed_c4789.docx [file pone.0326164.s001.docx]

**Reviewers' comments to editor:**

This manuscript describes "Protection of LPS-Induced Intestinal Injury in Goslings by

Polysaccharide of Atractylodes macrocephala Koidz Based on 16S rRNA and Metabolomics Analysis".

The topic is of interest. However, there are several concerns about the study, and needs to be improved. Major revision.

1-In the abstract section, the sample types of the two omics need to be supplemented.

2- The important detection indicators should be listed in the abstract, and the conclusions need to be further clarified

3- In the introduction section, lack of epidemiological data support, please add it.

4-It is not clear why metabolomics technology is applied to this research direction, and relevant literature needs to be supplemented to support this research theme.

5-In the section of materials and methods, no clear ethical certification number for animal experiments is provided, please add.

6- No animal qualification number is provided

7- Detailed sample collection process and purpose? Sample sizes used for different experiments should be specifically described.

8- The item numbers and test steps of different kit indicators should be supplemented, as well as the detection limits.

9- The details of 16s sequencing sample collection need to be supplemented.

10- 16s sequencing pathways and functional analysis procedures need to be supplemented

11- Non-targeted metabolomics steps are too simple and important steps need to be described

12- The upstream and downstream direction of primers should be clearly marked, see Table 2

13- The statistical analysis method needs to be further improved, and the difference of methods should be properly explained for some normal and skew data

14-Indicators that appear for the first time should be given full English names

In the results section, it is necessary to supplement the mass spectrometry of key markers and compare them with standard substances.

15- The remarks below all the chart are not detailed.

16- Important indicators such as differential multiples of metabolites (FDR) and VIP need to be listed.

17- The dominant flora should be written in italics

18- It is suggested that the results should be analyzed jointly with the two omics,

19- It is suggested that a joint analysis of the results of the two omics should be made, and that the discussion should fully discuss how metabolites regulate the flora and affect the intestinal function, and supplement the relevant literature.

20- In conclusion, the important metabolites and metabolic pathways as well as the changes of dominant flora should be highlighted

21-In the result part, it is necessary to supplement the mass spectrometry of key markers, and identify and compare with standard substances. At the same time, provide the structural formula of key markers found, VIP value and other key identification information

22-In the discussion section, it is necessary to supplement the information about the new experimental results, and to explore the metabolites, metabolic pathways and potential pathogenic mechanisms in depth.

Other comments：

1-It is suggested that references supplement the recent three years of research.

2-It is recommended that the article be polished by professionals or companies. There are currently problems with word writing or grammar.
