# Supplementary material for: Protection of LPS-induced intestinal injury in goslings by polysaccharide of Atractylodes macrocephala Koidz based on 16S rRNA and metabolomics analysis
Source: PLoS One. 2025 Jun 18;20(6):e0326164. doi: 10.1371/journal.pone.0326164 (PMC12176298; doi:10.1371/journal.pone.0326164)
Supplement: Attachment — Submitted filename: Response to Reviewer1.docx [file pone.0326164.s002.docx]

Reviewer #1: Reviewers' comments to editor:

This manuscript describes "Protection of LPS-Induced Intestinal Injury in Goslings by
Polysaccharide of Atractylodes macrocephala Koidz Based on 16S rRNA and Metabolomics Analysis".
The topic is of interest. However, there are several concerns about the study, and needs to be improved. Major revision.

1-In the abstract section, the sample types of the two omics need to be supplemented.

Thank you for your constructive comment. As per your suggestion, we have supplemented the abstract section with the sample types used for the two omics approaches. Specifically, the sample for 16S is cecal contents, and the sample for non-targeted metabolomics is serum. We hope this revision addresses your concern.

2- The important detection indicators should be listed in the abstract, and the conclusions need to be further clarified

Thank you for your valuable comments. We have carefully revised the abstract by incorporating the key detection indicators and further clarifying the conclusions to enhance the clarity and completeness of our findings. We hope that these changes meet your expectations. Please let us know if any further improvements are needed.

3- In the introduction section, lack of epidemiological data support, please add it.

Thank you for your insightful comments. We acknowledge the importance of incorporating epidemiological data to strengthen the background of our study. However, our research focuses on intestinal damage caused by the accumulation of LPS due to various factors rather than a specific bacterial or viral infection. Therefore, epidemiological data related to a single pathogen may not be directly applicable in this context.

To address your concern, we have revised the introduction to include relevant findings on the impact of LPS accumulation on intestinal health. Specifically, we have highlighted that LPS, as a major virulence factor of Gram-negative bacteria, can induce inflammation, disrupt the gut microbiota, impair intestinal barrier function, and contribute to metabolic disorders. Moreover, LPS accumulation has been linked to diarrheal diseases in various species, including poultry, as it promotes immune activation and intestinal fluid secretion. These additions underscore the broader significance of LPS-related intestinal dysfunction beyond specific infectious agents.

The revised content can be found in the updated manuscript (Lines 7–27). We appreciate your valuable feedback and hope that these modifications adequately address your concerns. Please let us know if any further refinements are needed.

4-It is not clear why metabolomics technology is applied to this research direction, and relevant literature needs to be supplemented to support this research theme.

Thank you for your valuable comment. We understand the importance of clarifying the rationale for applying metabolomics technology in this study. To address this concern, we have revised the manuscript to explicitly highlight the significance of metabolomics in studying gut microbiota function and intestinal health.

Specifically, we have elaborated on how metabolomics provides a comprehensive analysis of small-molecule metabolites, offering direct insights into microbial community functions and their regulatory effects on host metabolism. We have also included relevant literature demonstrating the successful application of metabolomics in inflammatory bowel disease research, where it has revealed disease-specific metabolic markers and pathway perturbations. Additionally, we have cited studies utilizing combined 16S rRNA sequencing and metabolomics approaches, showing how this integrative strategy has been instrumental in understanding gut microbiota-related metabolic mechanisms and developing potential therapeutic interventions.

These revisions can be found in the updated manuscript (Lines 41–67). We appreciate your thoughtful suggestion and believe that the added discussion strengthens the justification for using metabolomics in our study. Please let us know if further clarification is needed.

5-In the section of materials and methods, no clear ethical certification number for animal experiments is provided, please add.

Thank you for your comment. We acknowledge the importance of providing clear ethical certification details for animal experiments. In response to your suggestion, we have added the ethical approval information in the Materials and Methods section:

"The experiment received prior ethical approval in accordance with Zhongkai University of Agriculture and Engineering and under the approved protocol number ZK202110-10."

This revision can be found in the updated manuscript (Line 120). We appreciate your careful review and hope this addition adequately addresses your concern. Please let us know if any further clarification is needed.

6- No animal qualification number is provided

Thank you for your comment. We appreciate your attention to the ethical details of our study. In response to your suggestion, we have added the animal qualification number in the Materials and Methods section as follows:

"The experiment received prior ethical approval in accordance with Zhongkai University of Agriculture and Engineering and under the approved protocol number ZK202110-10, with animal qualification number NO20210906.."

This revision can be found in the updated manuscript (Line 121). We hope this addition addresses your concern. Please let us know if any further clarification is needed.

7- Detailed sample collection process and purpose? Sample sizes used for different experiments should be specifically described.

Thank you for your valuable comment. We understand the importance of clearly detailing the sample collection process and specifying the sample sizes used for different experiments. In response to your suggestion, we have revised the Materials and Methods section to include a comprehensive description of the sample collection process and its purpose, as outlined below:

1. Serum Sample Collection:
   - Sample Size: 10 randomly selected geese per group. 6 samples were randomly selected for Non-targeted metabolomics and 9 samples were randomly selected for ELISA.
   - Process: Blood was collected via cardiac puncture using disposable blood collection needles and stored in yellow-cap tubes containing coagulants and separation gel.
   - Purpose: Serum contains multiple inflammatory cytokines (IL-1β, IL-6, IL-18, TNF-α) and non-specific inflammatory markers (PCT, CRP). Their levels provide insight into the degree of systemic inflammation. Additionally, untargeted metabolomics was performed to comprehensively analyze metabolic changes in the serum, aiding in the identification of potential biomarkers and metabolic pathways related to LPS-induced intestinal damage.
2. Intestinal Tissue Collection (Jejunum):
   - Sample Size: Collected from the same geese after blood sampling and 7 samples were randomly selected for q-PCR.
   - Process: Geese were euthanized via intraperitoneal injection of pentobarbital sodium (100 mg/kg BW) and dissected under aseptic conditions. Jejunum tissue was excised 10 cm away from the ileocecal junction, washed with physiological saline, and stored in liquid nitrogen.
   - Purpose: The jejunum plays a crucial role in digestion and nutrient absorption. The collected samples were used for gene expression analysis, specifically evaluating inflammation-related genes (IL-1β, IL-6, IL-18, TLR-2) and tight junction proteins (ZO-1, Occludin, Claudin, Mucin-2) to assess gut barrier integrity.
3. Cecal Content Collection:
   - Sample Size: Collected from the same geese after tissue sampling and 6 samples were randomly selected for 16s rRNA sequence.
   - Process: The cecum was carefully removed, and its contents were collected using sterile scissors and forceps, avoiding contamination. Approximately 1.5 mL of cecal content per sample was transferred into sterile cryotubes and immediately frozen in liquid nitrogen.
   - Purpose: The cecal microbiota plays a critical role in gut health. 16S rRNA sequencing was conducted to analyze the microbial composition and diversity, providing insights into the interaction between gut microbiota and LPS-induced intestinal damage.

We appreciate your careful review and believe that these additions clarify our sample collection methodology and experimental design. Please let us know if further modifications are required.

8- The item numbers and test steps of different kit indicators should be supplemented, as well as the detection limits.

Thank you for your thoughtful comment. In response to your suggestion, we have included the product codes of the ELISA kits used for different indicators in the Materials and Methods section. Additionally, we would like to clarify that the ELISA test steps for each kit were performed according to the manufacturer’s instructions. Since the operation procedures are extensive, we have attached the relevant manuals in the supplementary materials for your reference.

The revised section now reads:

"IL-1β (product code: ZK-G7321), IL-6 (product code: ZK-G7318), IL-18 (product code: ZK-G7323), PCT (product code: ZK-G7512), CRP (product code: ZK-G7610), and TNF-α (product code: ZK-G7319) ELISA kits were purchased from Shenzhen Zike Biotechnology Company (Shenzhen, China). The ELISA test procedures followed the manufacturer's guidelines, and the relevant operation manuals are provided in the supplementary materials." These revisions can be found in the updated manuscript (Lines 129–133).

We hope this addition addresses your concern. Please let us know if any further clarification is needed.

9- The details of 16s sequencing sample collection need to be supplemented.

Thank you for your valuable suggestion. In response to your comment, we have supplemented the Materials and Methods section with more detailed information regarding the 16S sequencing sample collection process. The revised section now includes the following:

"Ten 28-day-old goslings per group were randomly selected for sample collection. In a sterile laboratory environment, all procedures were performed by personnel who had received specialized training in dissection and sample collection. Prior to the procedure, sterile surgical instruments, including scalpels, forceps, scissors, and cryotubes, were sterilized using high-temperature and high-pressure methods. The goslings were deeply anesthetized by intraperitoneal injection of a lethal dose of sodium pentobarbital (100 mg/kg BW) to ensure painless euthanasia. After euthanasia, the goslings were fixed in a supine position on a surgical table, and the abdominal area was disinfected with 75% alcohol. A sterile surgical incision was made along the midline of the abdomen to expose the internal organs. The cecum was carefully identified, and using sterile forceps, it was gently removed from the abdominal cavity while avoiding any loss of cecal contents. A small incision was made at one end of the cecum with sterile scissors, and the contents were gently expelled into sterile cryotubes (approximately 1.5 mL per tube). Any abnormal samples (e.g., due to unusual color or odor) were discarded and re-collected. The tubes were immediately placed in liquid nitrogen for rapid freezing. Six randomly selected samples from each group were used for 16S sequencing." Considering that this part of the content in the manuscript should not take up too much space, we have briefly summarized this content and updated it in the main text. These revisions can be found in the updated manuscript (Lines 139–146). We hope this additional information clarifies the sample collection process. Please let us know if any further clarification is needed.

10- 16s sequencing pathways and functional analysis procedures need to be supplemented

Thank you for your valuable comment. In response, we have supplemented the materials and methods section with additional details regarding the 16S sequencing pathways and functional analysis procedures. These additions provide a clearer understanding of the methodology used in our study. Considering that this part of the content in the manuscript should not take up too much space, we have briefly summarized this content and updated it in the main text. These revisions can be found in the updated manuscript (Lines 147–178). We hope this addition addresses your concern regarding the 16S sequencing pathways and functional analysis. Please let us know if further clarification is required.

11- Non-targeted metabolomics steps are too simple and important steps need to be described

Thank you for your constructive suggestion. In response to your comment, we have supplemented the Materials and Methods section with a more detailed description of the non-targeted metabolomics workflow. The revised section now includes the following information:

Sample preparation

Thaw the experimental sample at 4 ℃, vortex the sample for 1 min after thawing, and

mix evenly; Accurately transfer an appropriate amount of sample into a 2 mL centrifuge tube; Add 400 µL methanol (stored at -20 ℃) and vortex for 1 min; Centrifuge for 10 min at 12,000 rpm and 4 ℃,take all the supernatant transfer it to a new 2 mL centrifuge tube, concentrate and dry it; Add 150 µL of 2-chloro-l-phenylalanine (4 ppm) solution prepared with 80% methanol water (stored at 4 ℃) to re dissolve the sample ,filter the supernatant by 0.22 μm membrane and transfer into the detection bottle for LC-MS detection.

Liquid chromatography conditions

The LC analysis was performed on a Vanquish UHPLC System (Thermo Fisher Scientific, USA).Chromatography was carried out with an ACQUITY UPLC ® HSS T3 (150×2.1 mm, 1.8 µm) (Waters, Milford, MA, USA). The column maintained at 40 ℃. The flow rate and injection volume were set at 0.25 mL/min and 2 μL, respectively. For LC-ESI (+)-MS analysis, the mobile phases consisted of (C) 0.1% formic acid in acetonitrile (v/v) and (D) 0.1% formic acid in water (v/v). Separation was conducted under the following gradient: 0~1 min, 2% C; 1~9 min, 2%~50% C; 9~12 min, 50%~98% C; 12~13.5 min, 98% C; 13.5~14 min, 98%~2% C; 14~20 min, 2% C. For LC-ESI (-)-MS analysis, the analytes was carried out with (A) acetonitrile and (B) ammonium formate (5mM). Separation was conducted under the following gradient: 0~1 min, 2%A; 1~9 min, 2%~50%A; 9~12 min, 50%~98%A; 12~13.5 min, 98%A; 13.5~14 min, 98%~2%A; 14~17 min, 2%A.

Mass spectrum conditions

Mass spectrometric detection of metabolites was performed on Orbitrap Exploris 120 (Thermo

Fisher Scientific, USA) with ESI ion source. Simultaneous MS1 and MS/MS (Full MS-ddMS2 mode, data-dependent MS/MS) acquisition was used. The parameters were as follows: sheath gas pressure, 30 arb; aux gas flow, 10 arb; spray voltage, 3.50 kV and -2.50 kV for ESI(+) and ESI(-), respectively; capillary temperature, 325 ℃; MS1 range, m/z 100-1000; MS1 resolving power, 60000 FWHM; number of data dependant scans per cycle, 4; MS/MS resolving power, 15000 FWHM; normalized collision energy, 30%; dynamic exclusion time, automatic.

These revisions can be found in the updated manuscript (Lines 180–212). We believe this expanded description provides a clearer and more detailed explanation of the non-targeted metabolomics methodology. Please let us know if further refinements are needed.

12- The upstream and downstream direction of primers should be clearly marked, see Table 2

Thank you for your valuable feedback. In response to your comment, we have clearly marked the upstream (forward) and downstream (reverse) directions of the primers in Table 2. This revision ensures clarity and accuracy in the primer sequence information.

Please refer to Table 2 for the updated details. Let us know if any further modifications are required.

13- The statistical analysis method needs to be further improved, and the difference of methods should be properly explained for some normal and skew data

Thank you for your valuable feedback. In this study, we primarily used one-way Analysis of Variance (ANOVA) to process the data and assess the significant differences between the groups. ANOVA is a commonly used method for analyzing normally distributed data and has good statistical power. All data meet the normality assumption, and significant differences between the groups were observed. Therefore, we believe that one-way ANOVA is appropriate for this study.

Once again, thank you for your suggestion. Your feedback is of great importance in improving our research.

14-Indicators that appear for the first time should be given full English names
In the results section, it is necessary to supplement the mass spectrometry of key markers and compare them with standard substances.

Thank you for your suggestion. We have carefully reviewed the manuscript and ensured that all indicators appearing for the first time are presented with their full English names. The revised terms have been highlighted in red for clarity.

Please let us know if any further refinements are needed.

15- The remarks below all the chart are not detailed.

Thank you for your valuable feedback. We have carefully revised and expanded the remarks below all the charts to provide more detailed explanations. These modifications ensure clarity and accuracy in data interpretation.

Please let us know if further refinements are required.

16- Important indicators such as differential multiples of metabolites (FDR) and VIP need to be listed.

Thank you for your valuable suggestion. We have now included the 24 differential metabolic results between P-LPS and LPS in the main text (Table 4), along with key indicators such as the false discovery rate (FDR) and variable importance in projection (VIP) values for the differential metabolites. Due to the large volume of data, we have provided additional details for other groups in a supplementary file (Differential metabolites.excel) for your review. Please refer to the attached document for the complete information.

If you require further clarification or additional details, please feel free to let us know.

17- The dominant flora should be written in italics

Thank you for your suggestion. We have revised the manuscript to ensure that the names of dominant flora are written in italics, as required. The modifications have been made in the Results section.

Please let us know if any further adjustments are needed.

18- It is suggested that the results should be analyzed jointly with the two omics,

Thank you for your valuable suggestion. We have incorporated the joint analysis of 16S sequencing and non-targeted metabolomics results in the manuscript. The integrated findings have been added to the Results section, highlighting the correlation between gut microbiota composition and key metabolic pathways. This addition strengthens the interpretation of our results and provides a more comprehensive understanding of the underlying mechanisms. These revisions can be found in the updated manuscript (Lines 390-407).

Please let us know if any further refinements are required.

19- It is suggested that a joint analysis of the results of the two omics should be made, and that the discussion should fully discuss how metabolites regulate the flora and affect the intestinal function, and supplement the relevant literature.

Thank you for your insightful suggestion. In response, we have expanded the discussion to include a detailed joint analysis of the 16S sequencing and non-targeted metabolomics results. Specifically, we have discussed how key metabolites regulate gut microbiota composition and influence intestinal function. Additionally, we have supplemented the discussion with relevant literature to further support our findings. These revisions can be found in the Discussion section from line 498 to line 535.

We appreciate your valuable feedback and welcome any further suggestions for improvement.

20- In conclusion, the important metabolites and metabolic pathways as well as the changes of dominant flora should be highlighted

Thank you for your valuable suggestion. We have revised the conclusion section to highlight the key metabolites and metabolic pathways, as well as the changes in dominant flora. These modifications emphasize the core findings of our study and their significance. The revised conclusion can be found in the Conclusion section. These revisions can be found in the Discussion section from line 537 to line 548.

We appreciate your insightful feedback and welcome any further suggestions for improvement.

21-In the result part, it is necessary to supplement the mass spectrometry of key markers, and identify and compare with standard substances. At the same time, provide the structural formula of key markers found, VIP value and other key identification information

Thank you for your constructive feedback. In response to your suggestion, we have supplemented the results section with the mass spectrometry data of the key markers. We have also included comparisons with standard substances, as well as the structural formulas of the key markers identified. Additionally, we have provided the VIP values and other key identification information. These additions can be found in the Results section, from line 350 to line 357.

We appreciate your valuable input and hope the revisions meet your expectations.

22-In the discussion section, it is necessary to supplement the information about the new experimental results, and to explore the metabolites, metabolic pathways and potential pathogenic mechanisms in depth.

Thank you for your valuable suggestion. In response to your comment, we have supplemented the discussion section with relevant information regarding the new experimental results. We have also expanded the discussion to explore the metabolites, metabolic pathways, and potential pathogenic mechanisms in greater depth. These additions can be found in the Discussion section, from line 498 to line 535.

We appreciate your feedback and hope the revisions meet your expectations.

Other comments：
1-It is suggested that references supplement the recent three years of research.

Thank you for your suggestion. In response to your comment, we have supplemented the manuscript with relevant references from the past three years to ensure the research is up-to-date. These newly added references can be found throughout the manuscript.

We appreciate your constructive feedback and hope the revisions meet your expectations.

2-It is recommended that the article be polished by professionals or companies. There are currently problems with word writing or grammar.

Thank you for your suggestion. We have carefully followed your recommendation and had the manuscript professionally polished by a language editing service. The revised version has undergone thorough proofreading to correct any grammatical errors, improve clarity, and enhance readability.

Additionally, we have attached the proof of language editing for your reference.

We sincerely appreciate your valuable feedback, which has helped us improve the quality of our manuscript. Please let us know if further refinements are needed.

Thank you again for your time and consideration.
